# Loss of androgen receptor signaling in prostate cancer‐associated fibroblasts (CAFs) promotes CCL2‐ and CXCL8‐mediated cancer cell migration

**DOI:** 10.1002/1878-0261.12327

**Published:** 2018-07-10

**Authors:** Bianca Cioni, Ekaterina Nevedomskaya, Monique H. M. Melis, Johan van Burgsteden, Suzan Stelloo, Emma Hodel, Daniele Spinozzi, Jeroen de Jong, Henk van der Poel, Jan Paul de Boer, Lodewyk F. A. Wessels, Wilbert Zwart, Andries M. Bergman

**Affiliations:** ^1^ Division of Oncogenomics The Netherlands Cancer Institute (NKI) Amsterdam The Netherlands; ^2^ Division of Molecular Carcinogenesis The Netherlands Cancer Institute (NKI) Amsterdam The Netherlands; ^3^ Oncode Institute The Netherlands; ^4^ Division of Molecular Genetics The Netherlands Cancer Institute (NKI) Amsterdam The Netherlands; ^5^ Faculty of EEMCS Delft University of Technology Delft The Netherlands; ^6^ Division of Pathology The Netherlands Cancer Institute (NKI) Amsterdam The Netherlands; ^7^ Division of Urology The Netherlands Cancer Institute (NKI) Amsterdam The Netherlands; ^8^ Division of Medical Oncology The Netherlands Cancer Institute (NKI) Amsterdam The Netherlands

**Keywords:** androgen receptor, cancer cell migration, cancer‐associated fibroblasts, CCL2, CXCL8, EMT, prostate cancer

## Abstract

Fibroblasts are abundantly present in the prostate tumor microenvironment (TME), including cancer‐associated fibroblasts (CAFs) which play a key role in cancer development. Androgen receptor (AR) signaling is the main driver of prostate cancer (PCa) progression, and stromal cells in the TME also express AR. High‐grade tumor and poor clinical outcome are associated with low AR expression in the TME, which suggests a protective role of AR signaling in the stroma against PCa development. However, the mechanism of this relation is not clear. In this study, we isolated AR‐expressing CAF‐like cells. Testosterone (R1881) exposure did not affect CAF‐like cell morphology, proliferation, or motility. PCa cell growth was not affected by culturing in medium from R1881‐exposed CAF‐like cells; however, migration of PCa cells was inhibited. AR chromatin immune precipitation sequencing (ChIP‐seq) was performed and motif search suggested that AR in CAF‐like cells bound the chromatin through AP‐1‐elements upon R1881 exposure, inducing enhancer‐mediated AR chromatin interactions. The vast majority of chromatin binding sites in CAF‐like cells were unique and not shared with AR sites observed in PCa cell lines or tumors. AR signaling in CAF‐like cells decreased expression of multiple cytokines; most notably CCL2 and CXCL8 and both cytokines increased migration of PCa cells. These results suggest direct paracrine regulation of PCa cell migration by CAFs through AR signaling.

**Abbreviations**
AR: androgen receptor
CAF: cancer‐associated fibroblasts
EMT: epithelial–mesenchymal transition
PCa: prostate cancer
PCDF: prostate cancer‐derived fibroblasts
TME: tumor microenvironment

## 1 Introduction

Prostate cancer (PCa) is the second most common malignancy in men worldwide, associated with high morbidity and mortality and therefore a major health concern (Ferlay *et al*., 2015). There is an urgent need to dissect the pathophysiological mechanisms of PCa progression, which will enable the development of new treatment strategies. During the development of the prostate, epithelial cells depend on the stromal compartment for the maintenance of their homeostasis, while during carcinogenesis, stromal cells in the prostate tumor microenvironment (TME) show a malignant phenotype (Cunha *et al*., 1996). There is convincing evidence that the stromal cells in the TME play a key role in altering normal epithelial cells homeostasis which ultimately results in PCa development (Hayward *et al*., 1997). Key features of the TME are remodeling of the extracellular matrix, increased angiogenesis, and increased infiltration of protumoral immune cells (Rowley, 1998). The TME is mainly composed of extracellular matrix and nonmalignant stromal cells including smooth muscle cells, fibroblasts, pericytes, endothelial cells, and various resident and infiltrating immune cells (Gascard and Tlsty, 2016). These cells communicate with each other and epithelial cells via soluble mediators, such as cytokines, and intercellular receptor–ligand interactions (Ishii *et al*., 2016; Kalluri and Zeisberg, 2006; Tlsty and Coussens, 2006). Cancer‐associated fibroblasts (CAFs) represent a heterogeneous population of cells in the TME that are key players in stromal alterations, which might contribute to malignant degeneration and progression (Gascard and Tlsty, 2016).

The majority of primary PCas are adenocarcinomas expressing the androgen receptor (AR), a nuclear steroid hormone receptor critically involved in PCa development and progression (Heinlein and Chang, 2004). The importance of AR in PCa biology is underlined by the fact that abrogation of AR signaling by testosterone ablation is the most effective treatment of metastasized disease and is of value as an adjuvant treatment for definitive radiotherapy of the prostate (Labrie, 2011). However, not only the epithelial prostate cells express AR but also stromal cells (Singh *et al*., 2014). Lower levels of AR expression in the TME was associated with a higher malignancy grade (Gleason score) of the tumor, higher tumor stage, a higher disease recurrence rate after prostatectomy, and a shorter progression‐free survival of metastasized patients treated with testosterone ablation, which suggests a protective role of AR signaling in the TME against malignant transformation and disease progression (Henshall *et al*., 2001; Olapade‐Olaopa *et al*., 1999; Wikstrom *et al*., 2009). However, it remains elusive whether AR expression in CAF‐like cells is causally involved in observed clinical events. Furthermore, no mechanism is known by which such transeffect may occur.

In this study, we describe the genomic actions of AR signaling in CAF‐like cells and identify secreted factors that are critical for transregulating PCa cell migration.

## 2 Materials and methods

### 2.1 Patients included in the study, immunohistochemistry FFPE tissue, and digital scoring of stromal AR expression

The experiments were undertaken with the understanding and written consent of each patient included in this study. The study methodologies were conformed to the standards set by the Declaration of Helsinki and were performed in accordance with the Code of Conduct of the Federation of Medical Scientific Societies in the Netherlands.

For immunohistochemistry (IHC), 5‐μm sections of formalin‐fixed paraffin‐embedded (FFPE) prostate samples or cytospins were prepared. Hematoxylin and eosin (H&E) staining was performed according to standard protocols. IHC was performed using the BenchMark automated immunostainer and iView detection system (Ventana Medical System, Tucson, AZ, USA). Antibodies used are listed in Table S1. Two cohorts were formed of FFPE prostatectomy specimen of patients with a Gleason 7 and patients with Gleason ≥ 8 malignancy grade. These cohorts were matched for age, initial prostate‐specific antigen (PSA), and T‐stage and not associated with metastases (10 in total), using r software (The R Foundation, Vienna, Austria). Two other cohorts were formed of FFPE prostatectomy specimen of patients with untreated localized PCa and patients with PCa metastasized to the locoregional lymph nodes. These cohorts were matched for Gleason score, age, initial serum PSA, and T‐stage (20 in total). Core biopsies of cancer and normal prostate tissue, as identified by a pathologist, of all samples in the cohorts were included in a Tumor Micro Array and stained for AR. Pan‐cytokeratin staining was used for demarcating the border between the epithelium and stroma. ImageJ tools Color Deconvolution, Despeckle, and Watershed options were used for quantification of AR expression (Ruifrok and Johnston, 2001). A two‐tailed Student's *t*‐test was performed to compare stromal AR expression between the cohorts.

### 2.2 Generation of short‐term fibroblast‐like cell cultures, other cell cultures, cell proliferation assays, and scratch assays

Biopsies were taken directly after prostatectomy from locations of PCa identified by multiparametric MRI and palpation of the tumor and immediately transferred to the laboratory. Primary fibroblast cultures were established as previously described (Villegas and McPhaul, 2005). Fibroblasts could be cultured for a maximum of seven passages before they become senescent. CAF‐like cells were cultured in medium 1, and immortalized human fibroblast BJ‐htert, histiocytic lymphoma U937, PCa cells LNCaPs, CWR‐R1, and lung cancer cells SW1573 were cultured in medium 2, while PC346C was cultured in medium 3 (Table S2). Human PC346C PCa cells were a kind gift from WM van Weerden, Erasmus Medical Center, Rotterdam, the Netherlands (Marques *et al*., 2005).

To assess cell proliferation, human PCa LNCaP cells, CWR‐R1, and short‐term cultured CAF‐like cells were seeded in a 96‐well plate and placed in an incubator outfitted with an IncuCyte Zoom microscope (Essen Biosciences, Ann Arbor, MI, USA) with a 10× objective. Phase‐contrast pictures were taken every 4 h. The integrated analyzer within the incucyte zoom software (Incucyte, Sartorius, Essen Bioscience, Ann Arbor, MI, USA) calculated confluence.

For scratch assays, CWR‐R1 cells were cultured in six‐well plate in 10% FBS medium (Table S2). Once confluency was reached, cultures were scratched with a 200‐μL tip and 1 pg·mL^−1^ of CCL2 or CXCL8 cytokines or H_2_O control was added to the culture medium. Cell migration was measured at the 0‐ and 96‐h time point under a microscope (Leica DM4000B, Leica microsystems, Wetzlar, Germany). Alternatively, after scratching, medium was replaced with 1 : 1 FCS‐proficient RPMI medium and charcoal‐stripped [*N*,*N*′‐dicyclohexylcarbodiimide (DCC)] conditioned medium (CM) from CAF‐like cells. Migration of the cells was assessed at the 0 and 6 days after creating the scratch. Migration of cells was quantified using ImageJ and expressed as percentage of closure compared to control. LNCaP cells could not be used for scratch assays because they easily detached from the plates.

AR signaling in CAF‐like cells was activated by adding testosterone analogue R1881 (Sigma R0908, Sigma‐Aldrich, St Louis, MO, USA) to DCC medium at concentrations ranging 10^−7^–10^−9^ m. Antiandrogen RD162 (Axon MedChem, 1532) alone or in combination with R1881 was added to DCC medium at concentrations ranging 10^−5^‐10^−6^ m.

Duration of R1881/RD162 exposure was 4 or 24 h. After exposure, CAF‐like cells were washed vigorously with PBS and culturing was continued in drug‐free DCC medium for 24 h, whereafter the supernatant was removed and stored. DCC‐CM was used for IncuCyte experiments 1 : 1 with FCS‐proficient medium for cell growth assays and scratch assays.

CCL2 and CXCL8 (Sigma) were used at concentrations ranging from 10^−1^ to 10^2^ pg·mL^−1^ in FCS‐proficient medium for growth and scratch assays with CWR‐R1 PCa cells.

### 2.3 Subcellular fractioning and western blot analysis

Cells were scraped, spun down, and resuspended in PBS supplemented with protease cocktail inhibitor (Sigma). Subcellular fractioning was performed essentially as described (Mendez and Stillman, 2000).

For western blot, cultured cells and biopsies were lysed using lysis buffer (Merck Millipore, Amsterdam, the Netherlands) supplemented with protease cocktail inhibitor (Sigma) and used for western blot analysis using standard protocols. Antibodies used are listed in Table S1.

### 2.4 Chromatin immunoprecipitation analyses, Solexa sequencing, and ChIP‐seq data processing

Chromatin immunoprecipitation was performed as previously described (Schmidt *et al*., 2009; Stelloo *et al*., 2015). Antibodies used are listed in Table S1.

DNA was amplified using standard procedures, as described previously (Jansen *et al*., 2013). DNA was sequenced using an Illumina Hiseq 2500 Genome Analyzer with 65‐bp single‐end reads. Sequences were aligned to the reference human genome (Hg19, February 2009) and data processing was performed as previously described (Stelloo *et al*., 2015). Peak calling was performed using two algorithms: MACS 1.4 and DFilter. Only the peaks called by both algorithms were used for further analysis. Peaks present in at least one of two replicates were used to construct the list of peaks present in fibroblasts.

DNA copy number of prostate cancer‐derived fibroblasts (PCDFs) and prostate tumors was extracted from ChIP‐seq data with CopywriteR package, which was run with default parameters (Kuilman *et al*., 2015).

In addition, we analyzed the overlap of ChiP‐seq data with a multitude of transcription factors motifs using the ReMap annotation tool (Griffon *et al*., 2015). Motif analysis, genomic distributions of bindings sites, differential binding analysis, and integration with gene expression data were performed as previously described (Stelloo *et al*., 2015).

### 2.5 RNA isolation, TruSeq stranded mRNA sample preparation and sequencing and integration with ChiP‐seq data

Patient‐derived CAF‐like cells were stimulated with 10^−9^ m of R1881 alone or in combination with 10^−6^ m of RD162 or vehicle for 8 h. RNA isolation was performed with Trizol according to the manufacturer's instructions (Invitrogen, Thermos Scientific, Carlsbad, CA, USA).

Strand‐specific libraries were generated using the TruSeq Stranded mRNA sample preparation kit (Illumina Inc., San Diego, CA, USA; RS‐122‐2101/2) according to the manufacturer's instructions (Illumina, Part # 15031047 Rev. E). Libraries were analyzed on a 2100 Bioanalyzer using a 7500 chip (Agilent, Santa Clara, CA, USA), diluted, and pooled equimolar into a 12‐plex, 10 nm sequencing pool. The libraries were sequenced with 65 base single reads on a HiSeq2500 using V4 chemistry (Illumina Inc.).

Integration of ChIP‐seq and RNA‐seq data was performed using BETA tool available through Galaxy Cistrome (Chawla *et al*., 2013). As ChIP‐seq data input, we used a consensus list of AR binding sites under R1881 stimulation, while as transcriptomic input, we used the analysis of differential gene expression between R1881 stimulated fibroblasts and nonstimulated controls. Differential gene expression analysis was performed using *limma* R package. BETA was run with default parameters.

### 2.6 Cytokines array

Human prostate‐derived CAF‐like cells were cultured in DCC medium (Table S2) and stimulated for 8 and 24 h with 10^−9^ m of R1881 or vehicle. A customized Luminex assay (R&D Systems, LXSAHM, R&D Biosystems, Bio‐Techne, Minneapolis, MN, USA) was used to measure cytokines in CAF‐like cell medium according to the supplier's protocol. Antibody‐coated beads were specific for CXCL8, CCL2, IL‐34, CXCL5, and CXCL1 (selected based on fold change) (all provided in the kit).

### 2.7 Transwell migration and invasion assay

Ninety‐six transwell plates with 8 μm pore size (Corning, CLS3374‐2EA, Corning, NY, USA) were used to assess the migration and invasion ability of CWR‐R1 cells in the presence or absence of neutralizing CCL2 (R&D Systems, MAB279‐SP) and CXCL8 (R&D Systems, MAB208‐SP) antibodies in fibroblasts CM. CWR‐R1 cells were seeded on top of the transwell membrane. In the lower chamber CM from fibroblasts stimulated with DMSO, R1881 alone or in combination with RD162 was added 1 : 1 with FBS‐RPMI, in the absence or presence of anti‐CCL2 and anti‐CXCL8 antibodies (1 ng·mL^−1^). To assess invasion ability of CWR‐R1 cells, Matrigel (Sigma; E1270) was added on top of the membrane before CWR‐R1 cells were seeded. After 48 h, CWR‐R1 cells that migrated on the other side of the membrane were quantified using crystal violet.

## 3 Results

### 3.1 Levels of AR staining in PCa‐associated stromal cells is inversely correlated with Gleason score and metastatic disease

**Figure 1:**
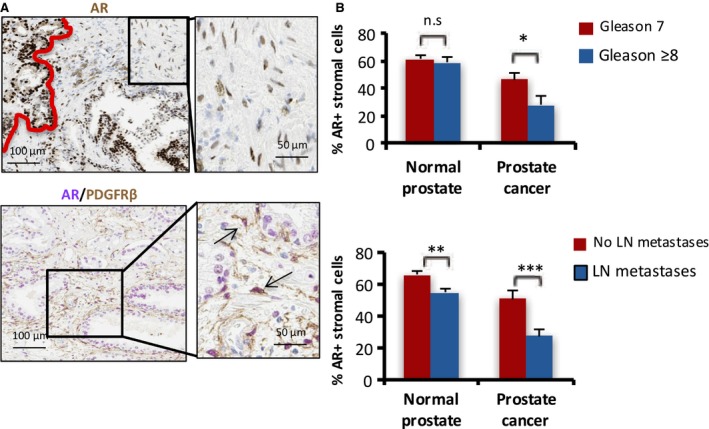
Stromal androgen receptor (AR) expression in PCas is associated with Gleason score and metastatic disease. (A) Immunohistochemistry staining for AR (nuclear; brown) in human PCa (left of the red boundary) and stroma (top). Double staining for AR (nuclear; purple) and the fibroblast marker PDGFRβ (cytosol; brown) (bottom). Insets show magnification of the stromal area. Arrows indicate PDGFβ‐positive fibroblasts with nuclear AR staining. (B) Percentage of AR‐positive cells in the tumor‐associated stroma and stroma in a healthy region of prostatectomies with tumors with a high (≥ 8) Gleason score, compared to tumors with an intermediate (7) Gleason score (top; *n* = 11; error bars represent SEM *P = 0.032). Percentage of AR‐positive cells in the tumor‐associated stroma and stroma in a healthy region of prostatectomies with tumors associated with metastases to locoregional lymph nodes compared to tumors without metastases (bottom; *n* = 19; error bars represent SEM; **P = 0.007, ***P = 0.004).

Androgen receptor is the key driver of PCa development and progression. AR staining is not only found in the epithelial compartment of human PCa specimens but also in stromal cells (Fig. 1A). Double staining for AR and the fibroblast marker PDGFRβ revealed that fibroblasts in the TME are AR‐expressing cells (Fig. 1A).

To assess any potential clinical implications of stromal AR levels, percentage of AR‐positive cancer‐associated stromal nuclei was compared between prostatectomy specimen with an intermediate Gleason score and a high Gleason score and between prostatectomy specimen associated with and without pelvic lymph node metastases. In line with previous reports (Olapade‐Olaopa *et al*., 1999; Wikstrom *et al*., 2009), a high Gleason score (≥ 8) was associated with a lower percentage of AR‐positive PCa‐associated stroma nuclei compared to a lower Gleason score, while no differences were found in normal stroma (Fig. 1B). A lower percentage of AR‐positive PCa‐associated stroma nuclei was observed in the primary tumors of patients with lymph node negative disease as compared to patients with pelvic lymph node metastases (Fig. 1B). There was also a small, but statistically significant difference found in normal prostate stroma (Fig. 1B). One of the possible explanations is that normal biopsies, despite being tumor‐negative, might still be affected by the TME.

Cumulatively, a high malignancy grade and the presence of lymph node metastases were associated with a lower AR expression in the PCa‐associated stroma.

### 3.2 Prostate cancer‐isolated fibroblasts are of mesenchymal lineage and retain AR expression

**Figure 2:**
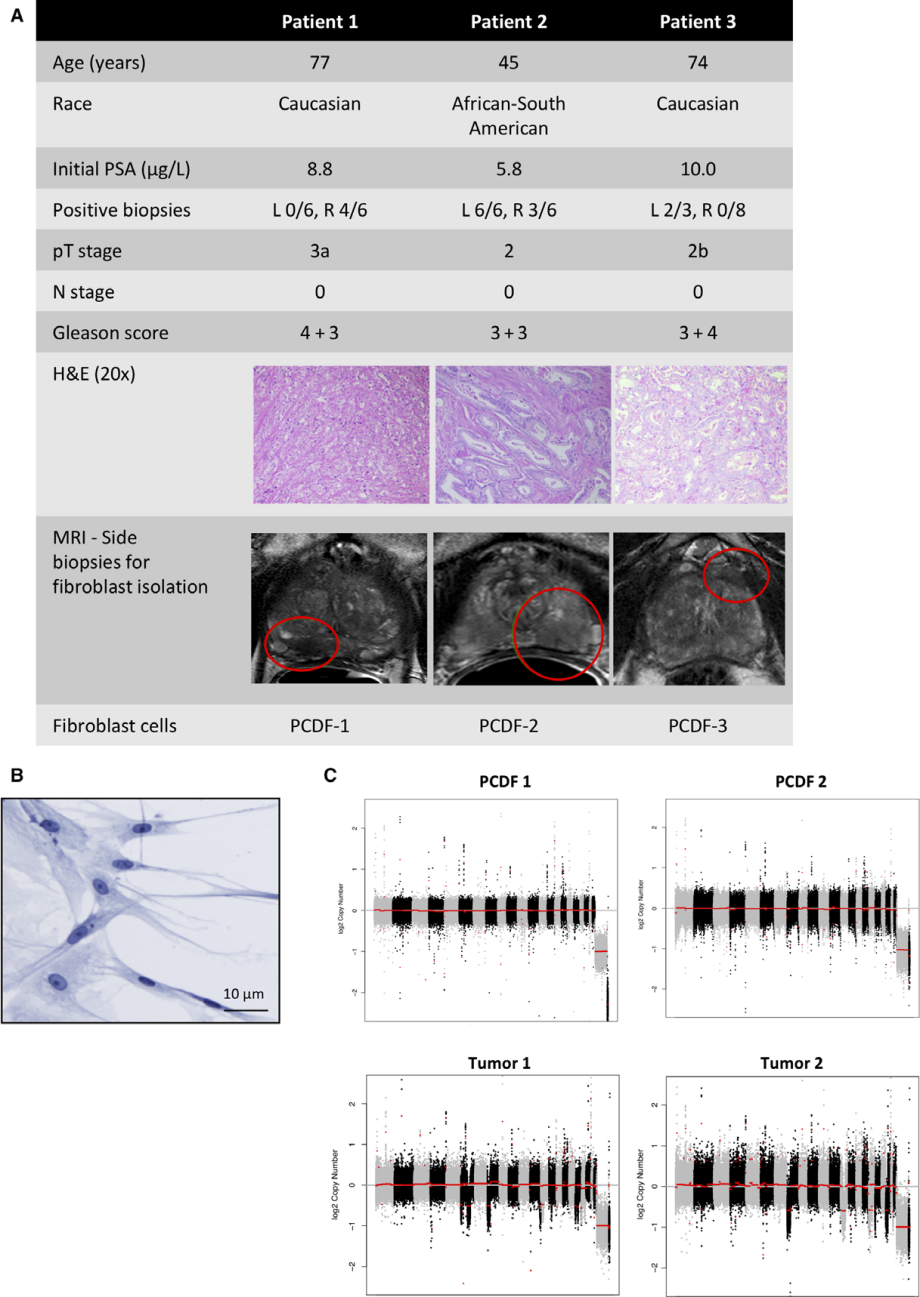
Patients and fibroblast characteristics. (A) Characteristics of the three patients of whom fibroblasts were cultured from biopsies of a cancer‐affected side of the prostates. Side selection for taking biopsies was based on the highest proportion of tumor‐containing diagnostic biopsies, multiparametric MRI images, and palpation of the tumor. (B) Representative phase‐contrast image of cells isolated from human PCa specimens shows fibroblast‐like morphology. (C) Copy number analysis in PCDF‐1 and PCDF‐2 cells (top) and two representative PCas (bottom).

In order to study AR signaling in fibroblasts in relation to PCa development, short‐term fibroblast cultures were established. Biopsies were taken from the cancer‐affected sites of three prostatectomy specimens: one with a low (3 + 3) and two with an intermediate (3 + 4 and 4 + 3) Gleason score. Other characteristics were as follows: patient age ranging from 45 to 77 years, T‐score ranging from 2 to 3a, initial PSA ranging from 5.8 to 10 μg/L, and all without local lymph node metastases (Fig. 2A). Biopsies were fragmented and monolayers of cells were established from the explants, which were cultured for up to seven passages. The cells derived from the biopsies showed a fibroblast‐like morphology, and cultures were designated as PCDF 1, 2, and 3 (Fig. 2A,B). Furthermore, DNA copy number profiling showed that fibroblasts were diploid throughout the genome compared to human PCas which harbored multiple DNA gains and losses (Fig. 2C).

**Figure 3:**
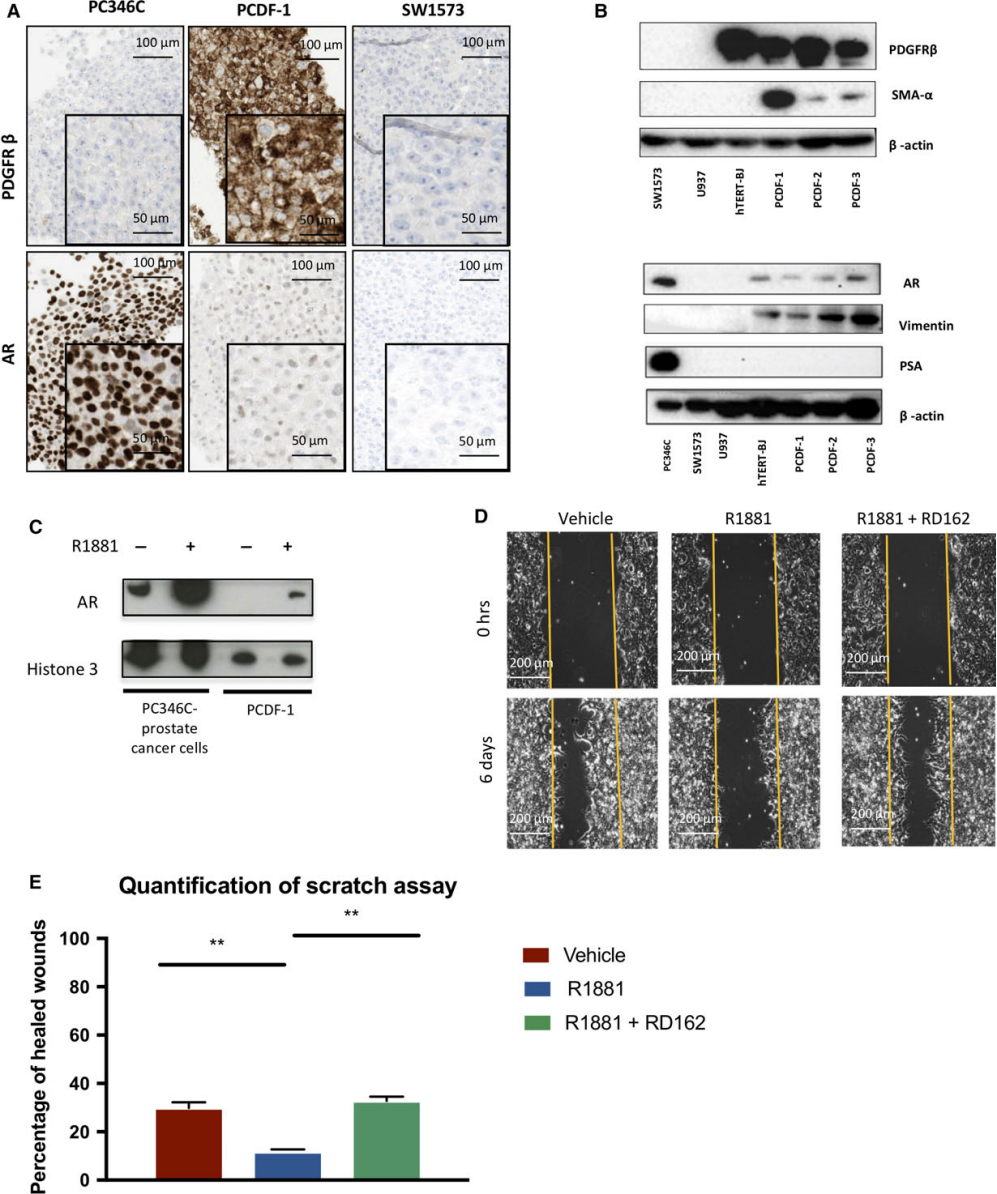
PCa‐derived fibroblasts have CAF‐like features and express functional AR. (A) Immunohistochemical staining for the fibroblast marker PDGFRβ (top) and AR (bottom), in PC346C PCa cells (left), PCa‐derived PCDF‐1 fibroblasts (middle), and SW1573 lung cancer cells. (B) Western blot for PDGFRβ, SMA‐α, AR, Vimentin, PSA, and β‐actin expression in SW1573 lung cancer cells, U937 histiocytic lymphoma cells, hTERT‐BJ fibroblasts and PCDF 1, 2, 3 cells. (C) Chromatin fractionation of hormone‐deprived PC346C PCa cells and PCDF‐1 fibroblasts, treated for 4 h with R1881 or DMSO control, and AR is stained. Histone 3 is used as loading control. (D) Scratch assay in human PCa CWR‐R1 cells. Cells cultured in CM of CAF‐like cells stimulated with vehicle, R1881 alone, or R1881 in combination with RD162. (E) Quantification of the scratch assay. Error bars show standard deviation of three replicates. Percentage of repopulation of the scratch surface after 96 h of culturing. ** represents *P* < 0.01.

PCDF cells stained positive for the fibroblast markers PDGFRβ, which was in contrast to human PCa PC346C cells, and human lung cancer SW1573 cells (Fig. 3A). AR‐positive staining was found in human PCa PC346C cells, and at a lower level in PCDF‐1 cells, while AR staining was absent in human lung cancer SW1573 cells (Fig. 3A).

The mesenchymal origin of the stromal cell cultures was further confirmed by western blot analyses, where, in contrast to PCa PC346C cells, the PCDF cells stained positive for the mesenchymal markers Vimentin and PDGFRβ, which was shared with the human telomerase‐immortalized foreskin fibroblast hTERT‐BJ1 (Fig. 3B). However, in contrast with the hTERT‐BJ1 fibroblasts, PCDF cells expressed SMA‐α, which suggests that these cells have CAFs features. AR expression was found in all PCDFs and in hTERT‐BJ1 cells, while PSA was uniquely found in PC346C PCa cells (Fig. 3B).

Cumulatively, these data show that the PCDF cells are of mesenchymal cell lineage but are not the result of epithelial to mesenchymal transition of PCa cells. Moreover, PCDF cells express AR and have CAF‐like features.

### 3.3 AR signaling in CAF‐like cells affects prostate cancer cell migration mediated by soluble factors

In PCa cells, the AR translocates to the nucleus upon R1881 and binds the chromatin to regulate expression of genes, ultimately leading to increased proliferation. Using subcellular fractionation assays, we found AR in CAF‐like cells also to bind the chromatin upon testosterone (R1881) stimulation, which suggests functionality of AR (Fig. 3C). However, R1881 stimulation did not alter the proliferation rate of PCDF cells (Fig. S1A) and also morphology and motility of these cells were not altered (data not shown). LNCaP PCa cell growth rate was not altered when culturing them in medium of PCDF cells stimulated with R1881 (Fig. S1B), suggesting that AR activation in PCDFs does not affect proliferation of PCa cells by soluble mediators.

As the presence of local PCa lymph node metastases was associated with a lower expression of AR in the stroma, we then speculated that AR signaling in PCDFs might affect PCa cell migration. In a scratch assay, migration of human PCa CWR‐R1 cells was inhibited by culturing in medium of PCDFs stimulated with R1881. This effect was reversed by coexposure to the AR signaling inhibitor RD162, suggesting a soluble mediator under control of AR signaling inhibits PCa cell migration (Fig. 3D,E).

Cumulatively, AR in CAF‐like cells binds the chromatin upon testosterone exposure, but does not affect CAF proliferation, morphology, or migration. However, our results suggest a direct transregulation of PCa cell migration by CAF‐like cells through AR signaling.

### 3.4 Androgen receptor occupies distinct chromatin sites in CAF‐like cells as compared to prostate cancer cells

**Figure 4:**
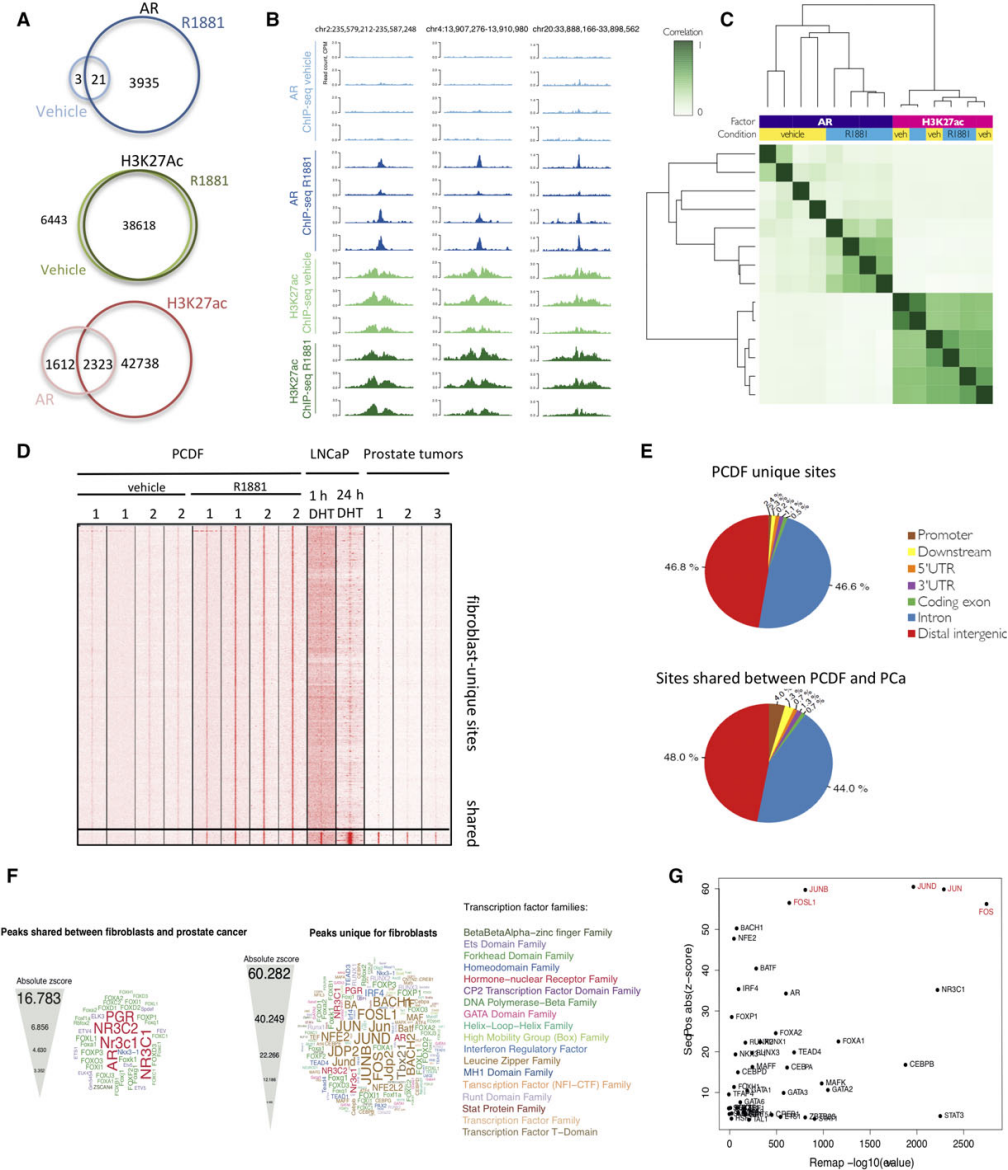
Androgen receptor occupies distinct chromatin sites in PCa‐derived fibroblasts. (A) Venn diagrams depicting overlap of AR (top) and H3K27Ac (bottom) binding sites in PCa‐derived fibroblasts, under vehicle and R1881 conditions. (B) Genome browser snapshot of AR and H3K27Ac ChIP‐seq in PCa‐derived fibroblasts. (C) Correlation heatmap of AR and H3K27Ac peaks, using supervised hierarchical clustering. (D) Heatmap depicting AR binding sites in PCa‐derived fibroblasts, LNCaP cells, and prostate tumors. All AR sites found in fibroblasts shown, grouped into ‘fibroblast‐unique’ and ‘shared’ sites, overlapping with LNCaP and PCa cells. Data are centered on the top of the AR peak within a 5‐kb window, where all data are vertically aligned. (E) Genomic distributions of AR binding sites relative to the most‐proximal gene, unique for fibroblasts (top), or shared between fibroblasts and PCa cells (bottom). (F) Motif analyses for the two separate AR peak subsets. Shared between fibroblasts and PCa cells (left) and unique for fibroblasts (right). (G) Scatter plot depicting enrichment scores for transcription factor overlap with ReMap analysis and scores from motif analysis.

To determine the genomewide chromatin profiles of AR in fibroblasts, we performed chromatin immunoprecipitation followed by massive parallel sequencing (ChIP‐seq). Therefore, PCDF1 and PCDF2 were hormone‐depleted for 3 days and subsequently treated for 8 h with R1881 or DMSO control, fixed, and processed for ChIP‐seq. AR is typically considered an enhancer‐selective transcription factor (He *et al*., 2007), where functional enhancers are hallmarked by H3K27Ac signals (He *et al*., 2010). Therefore, AR ChIP‐seq was followed by H3K27Ac ChIP‐seq in the same samples. Under DMSO conditions, 24 AR binding sites were found which increased to 3956 sites under R1881 conditions (Fig. 4A). While AR chromatin binding was induced by R1881, as expected, H3K27Ac signal was completely hormone independent, as exemplified in Fig. 4B. The number of H3K27ac peaks under DMSO conditions (6443) did not significantly alter after R1881 exposure (5723) (Fig. 4A). Approximately 60% of AR binding sites coincided with H3K27ac peaks (Fig. 4A). These results were also visualized by unsupervised hierarchical clustering analyses of correlations between peaks, where AR peaks under DMSO conditions grouped separately from those in the presence of R1881, while no such separation was observed for H3K27Ac (Fig. 4C).

As AR genomics is classically studied in the context of PCa cells, we next determined overlap of AR sites between PCDFs and PCa cells and tumors (Fig. 4D). Interestingly, the vast majority of AR sites found in PCDFs, and induced by R1881, were unique for this cell type and not shared with those found in prostate tumors and the PCa cell line LNCaP. Only a minor set of AR sites (260 sites) was shared between PCDFs and PCa cells. Genomic locations of AR sites did not deviate between fibroblast‐unique sites and those AR sites observed in PCDFs and PCa cells, with the vast majority of AR binding observed in distal intergenic regions and introns, which is a typical feature of enhancers (Fig. 4E). For both peak subsets, motifs for AR and forkhead transcription factors were found enriched (Fig. 4F), with Fos and Jun motifs strongly enriched in the PCDF‐unique AR sites. Functionality of the observed motifs for Fos and Jun was confirmed using meta‐data from the ReMap tool, indicating functional enrichment of JUND, JUN, and FOS at PCDF‐unique AR binding sites (Fig. 4G). This suggests that in PCDF‐like cells, AR binds the DNA via the AP1 complex of cofactors as described previously (Leach *et al*., 2017).

Next, both peak sets were coupled to the most‐proximal genes with a transcription start site within 20 kb from the most‐proximal AR site, or with an AR site within the gene body. These gene sets were subsequently assessed using ingenuity pathway analysis (IPA) (Fig. S2). The dominant biological processes in CAF‐like cells regulated by AR signaling were cell movement and migration, which were not found enriched in PCa cells.

Cumulatively, AR in CAF‐like cells binds the chromatin at distal intergenic regions and introns upon testosterone stimulation, presumably via the AP1 complex. The vast majority of binding sites are unique to CAF‐like cells and not shared with PCa cells and tumors.

### 3.5 RNA‐seq data combined with ChIP‐seq data identify CCL2 and CXCL8 as cytokines regulated by AR signaling in fibroblast‐like cells

**Figure 5:**
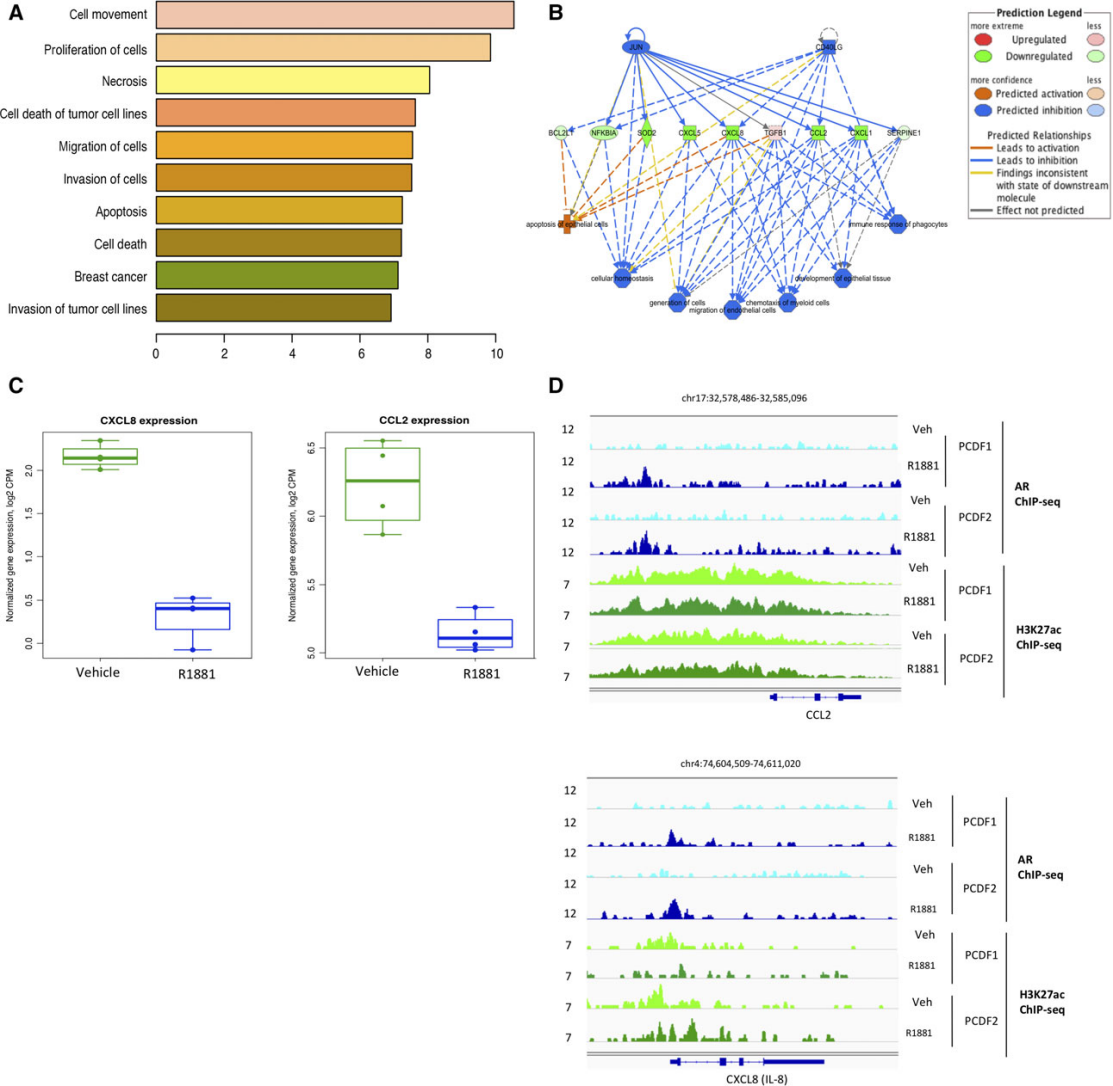
Effect of AR actions on gene expression. (A) Gene ontology terms analysis for biological process of differentially expressed genes in CAF‐like cells upon testosterone stimulation. (B) Ingenuity pathway analyses of differentially expressed genes suggest decreased expression of cytokines critically involved in cancer cell functions, such as cell migration and chemotaxis. (C) mRNA downregulation of CCL2 and CXCL8 upon R1881 stimulation. (D) AR and H3K27ac binding sites in CCL2 and CXCL8 gene regions. AR shows specific bindings upon R1881 stimulation.

To identify potential direct gene targets of AR in PCDFs, we employed integration of ChIP‐seq and transcriptomic (RNA‐seq) data. Using BETA analysis, we identified 174 genes that are potentially directly upregulated (Table S3) and 234 potentially directly downregulated genes (Table S4) by AR activation in PCDFs. Examples of upregulated targets include known AR targets, such as FKBP5 and DUSP1. Downregulated target genes include notable examples of immune‐related molecules CXCL8, CCL2, NFKBIA, and others. Functional analysis of these up‐ and downregulated target genes by IPA identified that these genes belong to networks typically regulated by TNF, HIF1A, JUN, IL‐17F, and IL‐1B (Fig. S3) and the molecular functions of these genes include cell movement, proliferation, and migration (Fig. 5A). The top regulator effect network identified JUN and CD40LG as the possible upstream regulators, inhibiting expression of cytokines, including CXCL5, CXCL8, CCL2, CXCL1, and IL‐34 (Fig. 5B), which regulate migration, chemotaxis, and immune response. Based on BETA analysis ranking, CCL2 and CXCL8 were the top two targeted genes downregulated in R1881 conditions compared to vehicle (Table S4, Fig. 5C). Figure 5D shows that AR binds the DNA upon R1881 stimulation in the proximity of the CCL2 and CXCL8 loci, further confirming transcriptional regulation of these two genes via AR binding.

**Figure 6:**
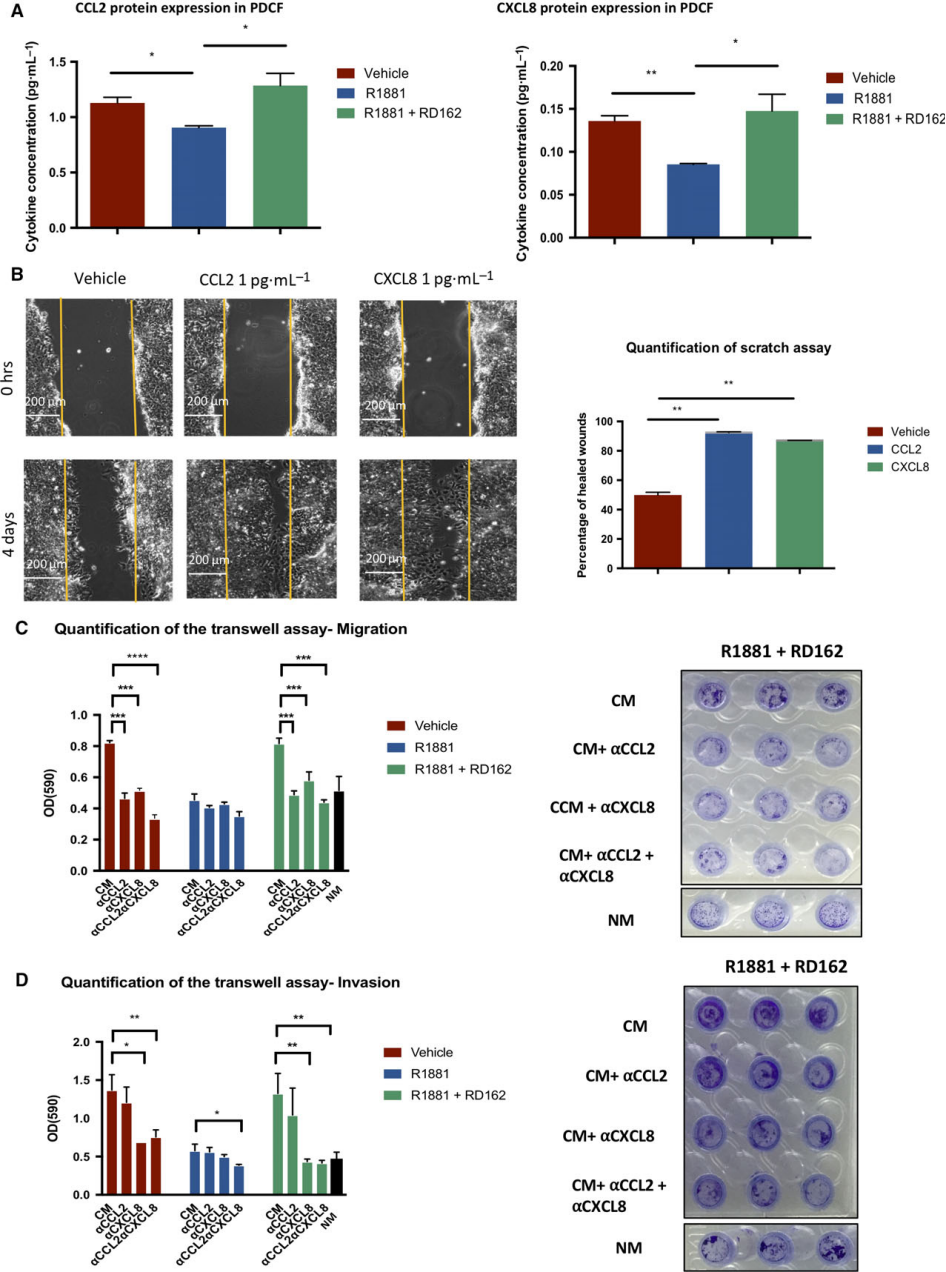
AR signaling in fibroblasts reduces PCa cell migration. (A) Decreased CCL2 and CXCL8 at the protein level upon R1881 stimulation for 24 h. Addition of RD162 restored the levels to unexposed cells, suggesting an AR signaling‐dependent regulation of cytokine expression. Average of three experiments. Error bars show standard deviation. (B) Scratch assay in CWR‐R1 cells. Addition of CCL2 or CXCL8 cytokines strongly increased cell migration at 1 pg·mL^−1^ (left). Quantification of the scratch assay (right). Percentage of repopulation of the scratch surface after 96 h of culturing. Error bars show standard deviation of three replicates. (C) Transwell migration assay. The migration of CWR‐R1 cells induced by fibroblast‐CM was reduced when αCCL2‐ and/or αCXCL8‐neutralizing antibodies were added in the lower chamber of the transwell. Normal medium (NM) was used as control. A representative of two independent experiments is shown. Error bars show standard deviation, and *, **, ***, **** represents *P* value < 0.05, < 0.01, < 0.001, < 0.0001, respectively. (D) Transwell invasion assay. The invasion of CWR‐R1 cells induced by fibroblast‐CM was reduced when αCCL2‐and/or αCXCL8‐neutralizing antibodies were added in the lower chamber of the transwell. Normal medium (NM) was used as control. A representative of two independent experiments is shown. Error bars show standard deviation, and *, **, ***, **** represents *P* value < 0.05, < 0.01, < 0.001, < 0.0001, respectively.

Using a customized Luminex kit, we then measured the protein expression level of CCL2, CXCL8, and other cytokines found to be downregulated at the RNA level upon R1881 stimulation (IL‐34, CXCL5, and CXCL1). Expression levels of the cytokines were measured in medium of PCDFs stimulated with R1881 or vehicle for 24 h. As shown in Fig. 6A, expression of CCL2 and CXCL8 was significantly downregulated upon testosterone stimulation compared to vehicle and production could be rescued by addition of antiandrogen RD162. IL‐34, CXCL5, and CXCL1 levels were very low and no difference in protein expression levels of these cytokines was observed between R1881 and vehicle‐treated cells (data not shown).

All together, these data suggest that CCL2 and CXCL8 expressions in PCDFs were directly downregulated by AR signaling both at RNA and at protein level.

### 3.6 Blocking AR signaling in CAF‐like cells increases prostate cancer cell migration mediated by increased secretion of CCL2 and CXCL8

To explore the effect of CCL2 and CXCL8 cytokines on PCa cells growth, LNCaP and CWR‐R1 cell lines were cultured in FCS‐proficient medium with addition of 1 pg·mL^−1^ of CCL2 or CXCL8, but only a marginal effect in reducing CWR‐R1 cell growth was seen (Fig. S4).

AR signaling in PCDFs inhibited migration of PCa cells via soluble mediators (Fig. 3D). To evaluate whether CCL2 and CXCL8 are mediators affecting PCa cell migration, migration of CWR‐R1 cells was measured in the presence of 1 pg·mL^−1^ of CCL2 or CXCL8. Pictures were taken every day. After 96 h, the percentage of repopulation of the scratch surface was compared to time point 0 and quantified. As shown in Fig. 6B, both cytokines strongly promoted PCa cell migration as compared to vehicle. As no stimulating effect of the two cytokines was observed in PCa cell growth (Fig. S3), we conclude that the cytokines affect migratory behavior of CWR‐R1 cells.

These results were validated using a transwell assay in which we assessed CWR‐R1 cell migration (Fig. 6C) and invasion (Fig. 6D), when cultured in fibroblast‐CM. Addition of anti‐CCL2, anti‐CXCL8, or both neutralizing antibodies in fibroblast‐CM blocked the promigratory effect on PCa cells mediated by AR‐blockade in fibroblasts (Fig. 6C and Fig. S5A). Furthermore, invasion ability of CWR‐R1 cells was also reduced in the presence of neutralizing anti‐CXCL8 antibodies (Fig. 6D and Fig. S5B).

All together these data show that decreased expression of CCL2 and CXCL8 in testosterone‐stimulated PCDFs reduces migration of PCa cells in the *in vitro* setting.

## 4 Discussion

The stromal microenvironment has emerged as a key player in the development and progression of cancer (Hanahan and Weinberg, 2011). During carcinogenesis, the composition of the stroma changes, characterized by a loss of well‐differentiated smooth muscle cells and appearance of so‐called myofibroblasts (Tuxhorn *et al*., 2002). Activated myofibroblasts, or CAFs are the principal components of the PCa microenvironment and are involved in PCa cell growth and invasion (Leach and Buchanan, 2017; Tuxhorn *et al*., 2002). We established short‐term cultures of prostate CAFs with CAF‐like features. DNA copy profiling of CAF‐like cells showed a normal, nonmalignant profile confirming that cells are from mesenchymal lineage and cancer cells are not a source of CAFs.

In agreement with others (Olapade‐Olaopa *et al*., 1999; Wikstrom *et al*., 2009), we report that AR is expressed in the PCa stroma and that levels of AR staining in the stroma are inversely related with the malignancy grade of the tumor and presence of pelvic lymph node metastases. Stromal cells expressing AR, such as CAFs, might undergo clonal selection upon pressure of AR action, or limited ligand availability during androgen deprivation therapy might lead to destabilization of less AR sensitive cells, such as stromal cells (Leach and Buchanan, 2017). Alternatively, epigenetic regulation could also be involved, as alterations in methylation state are known to control AR expression (Keil *et al*., 2014).

Despite the clear relationship between poor outcome and loss of stromal AR, the underlying mechanism involving AR signaling in CAFs and consequences in cancer progression and outcome remains largely unknown. Several mechanisms have been proposed, including AR‐regulated secretion of factors by CAFs, affecting PCa cell proliferation, and modification of the extracellular matrix (Leach and Buchanan, 2017). Here, we present an unbiased, genomic, and functional assessment of AR actions in human prostate‐derived CAF‐like cells in relation to PCa behavior.

Testosterone stimulation of CAF‐like cells did not affect morphology, motility, and proliferation, which was in contrast to observations in immortalized CAFs (Leach *et al*., 2015). We observed that CM of testosterone‐stimulated CAF‐like cells reduced migration of PCa cells, while antiandrogens restored tumor cell migration. As previously described in CAFs, we also observed AR binding in functional enhancer regions that are marked by the H3K27Ac acetylation mark and very limited overlap of AR sites was observed between fibroblasts, prostate tumors, and LNCaP (Nash *et al*., 2017; Nevedomskaya *et al*., 2016). We confirmed previous data showing that AR binding in human prostate CAFs might not only be dependent on the classic AR pioneer transcription factors such as FOXA, but rather act via the AP1 complex (Leach *et al*., 2017). This might suggest that AR in CAFs controls different biological processes compared to epithelial cells.

Here, we identified CCL2 and CXCL8 as key players in CAF‐mediated PCa cell migration and invasion using an unbiased and genomewide approach. Stimulation of CAFs with testosterone resulted in AR chromatin binding at CCL2 and CXCL8 loci, subsequently leading to significant downregulation of CCL2 and CXCL8 both at the mRNA and at protein level. Importantly, we showed that blocking antibodies targeting CCL2 and CXCL8 fully abrogated migration and invasion of PCa cells cultured in CM of AR signaling inhibited fibroblasts. These results indicate a direct effect of AR‐regulated cytokines on PCa cell behavior.

Our unbiased approach leads to the identification of secreted cytokines, suggesting that secreted factors mediate the effects on PCa cell migration. In addition, direct cell–cell contacts between fibroblasts and tumor cells may play an important role in the epithelial–stromal interaction as well and could be relevant mechanisms to explore in future work. In fact, previous studies show that CAFs are able to promote directional migration of PCa cells by aligning the fibronectin in the extracellular matrix (Erdogan *et al*., 2017). Furthermore, in coculture experiments, PCa cells were shown to be able to modulate the fibroblast–cancer cells interaction via deregulation of proteoglycans and junction molecules, impairing the interconnection with fibroblasts and facilitating migration (Suhovskih *et al*., 2017). The ability of CAFs to modulate PCa progression via cell–cell contact mechanisms was also confirmed *in vivo* in tissue recombinant mouse models (Olumi *et al*., 1999).

A relation between AR signaling and regulation of CCL2 expression was previously described in macrophages (Izumi *et al*., 2013). CCL2, also known as monocyte chemoattractant protein 1 (MCP1), is a strong chemoattractant for immune cells and is produced by a variety of different cell types (Deshmane *et al*., 2009). CCL2 was linked with PCa progression through macrophage recruitment and PCa cell migration (Lin *et al*., 2013; Wu *et al*., 2017). CXCL8, also known as IL‐8, is a chemokine that modulates cancer cell proliferation, invasion, and migration of multiple cancers (Liu *et al*., 2016). Multiple studies associated the expression of CXCL8 with poor clinicopathological features including poor differentiation, advanced tumor stage, cancer cell proliferation, and angiogenesis (Araki *et al*., 2007; Armstrong *et al*., 2016; Maxwell *et al*., 2013; Uehara *et al*., 2005). In mouse models of PCa, CXCL8 signaling was shown to promote the proliferation and invasion of PCa cells. (Inoue *et al*., 2000; Kim *et al*., 2001; Seaton *et al*., 2008). Release of CXCL8 by PTEN‐deficient PCa cells increased the expression of CCL2 and CXCL12 in stromal cells, which promoted human PC3 PCa cells migration (Maxwell *et al*., 2014). Moreover, CXCL8 stimulation of PCa cells was described to regulate cyclin D1 expression, supporting cell cycle progression and PCa tumor growth (MacManus *et al*., 2007).

In contrast to the effect of AR signaling inhibition on PCa proliferation, inhibiting AR signaling in CAFs might enhance PCa cell migration, as was suggested previously by Lin *et al*. (2013). Therefore, specific inhibitors of PCa AR, not affecting stromal cell AR might enhance antihormonal treatment efficacy. Alternatively, cell‐specific genes downstream of AR signaling might be targeted to selectively block AR‐mediated effects on PCa cells. Migration and invasion of PCa cells might be reduced by combining hormone therapy with blocking antibodies specifically targeting CCL2 and CXCL8 cytokines.

## 5 Conclusions

In conclusion, the present study showed how inhibition of AR signaling in fibroblast by hormone therapy might lead to unwanted effects on PCa development. This would suggest that classic hormonal therapies should be combined with targeted endocrine agents to improve efficacy.

## Author contributions

BC, EN, MHMM, AMB, and WZ conceived and designed the project; BC, EN, JvB, and AMB wrote the manuscript; BC, MHMM, EH, DS, JvB carried out the experiments; EN and SS performed data analysis and interpretation; HGvdP and JdJ provided specimens material and data interpretation. All the coauthors critically reviewed the present manuscript before submission.

## Supporting information


**Fig. S1.** Proliferation curves of PCDFs and PCa cells.
**Fig. S2.** Ingenuity Pathway Analysis of genes proximal to AR binding sites.
**Fig. S3.** Upstream regulators of R1881‐stimulated genes in PCDFs.
**Fig. S4.** CCL2 and CXCL8 effect on PCa cells proliferation.
**Fig. S5.** Migration and invasion assay.
**Table S1.** Antibodies list.
**Table S2.** Culture media conditions.
**Table S3.** R1881‐specific upregulated genes found with BETA analysis.
**Table S4.** R1881‐specific downregulated genes found with BETA analysis.
