# Supplementary material for: Loss of androgen receptor signaling in prostate cancer‐associated fibroblasts (CAFs) promotes CCL2‐ and CXCL8‐mediated cancer cell migration
Source: Mol Oncol. 2018 Jul 10;12(8):1308–23. doi: 10.1002/1878-0261.12327 (PMC6068356; doi:10.1002/1878-0261.12327)
Supplement: Supplementary file 1 — Fig. S1. Proliferation curves of PCDFs and PCa cells. Fig. S2. Ingenuity Pathway Analysis of genes proximal to AR binding sites. Fig. S3. Upstream regulators of R1881‐stimulated genes in PCDFs. Fig. S4. CCL2 and CXCL8 effect on PCa cells proliferation. Fig. S5. Migration and invasion assay. Table S1. Antibodies list. Table S2. Culture media conditions. Table S3. R1881‐specific upregulated genes found with BETA analysis. Table S4. R1881‐specific downregulated genes found with BETA analysis. [file MOL2-12-1308-s001.pdf]

Figure S1. Proliferation curves of PCDFs and PCa cells

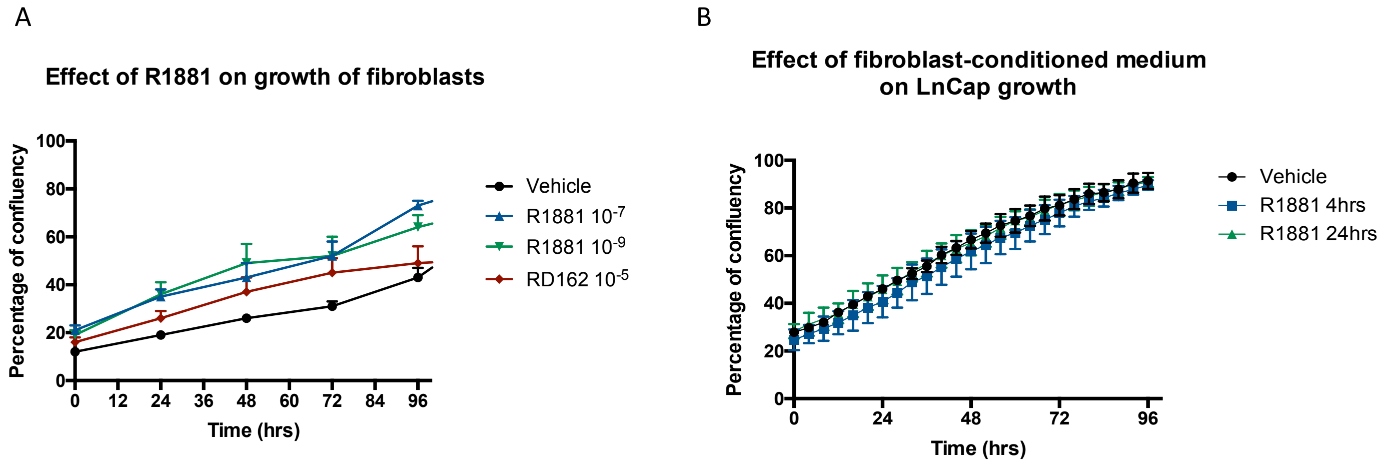

Supplementary Figure S1. A) Proliferation of prostate derived PCDF-1 fibroblasts was not affected by stimulation with R1881 alone or in combination with AR inhibitor RD162. Average of 3 experiments. Error bars indicate standard error of the mean. B) Proliferation of prostate cancer LNCaP cells was not affected by culturing in conditioned medium of CAF-like cells stimulated with R1881 for 4hrs and 24hrs. Average of 3 experiments. Error bars indicate standard error of the mean.

Figure S2. Ingenuity Pathway Analysis of genes proximal to AR binding sites

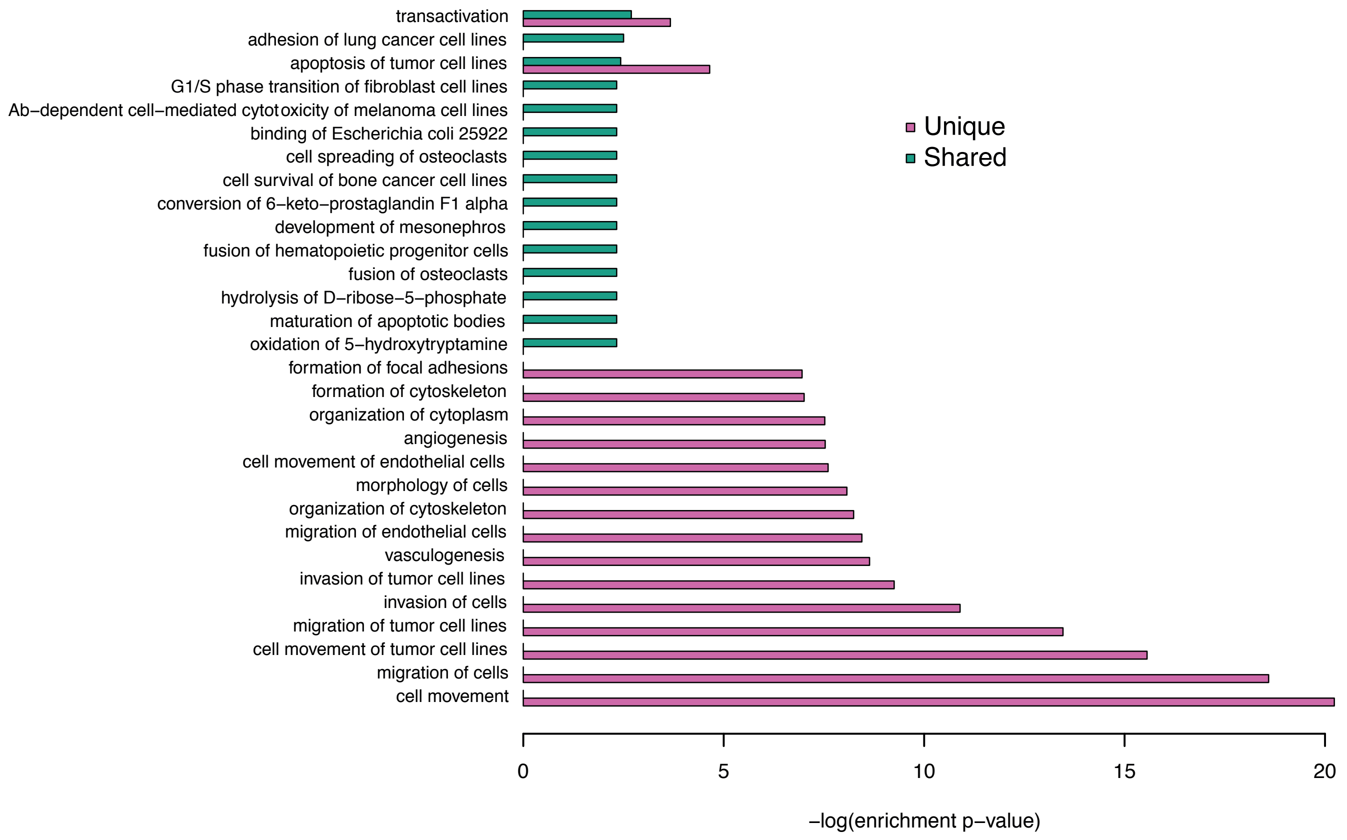

Supplementary Figure S2. Ingenuity Pathway Analysis for genes with a proximal (<20kb) AR binding sites uniquely found in CAF-like cells or shared between CAF-like cells and prostate cancer cells. Biological process enrichment is shown, (-log) p value is depicted.

Figure S3 Upstream regulators of R1881-stimulated genes in PCDFs

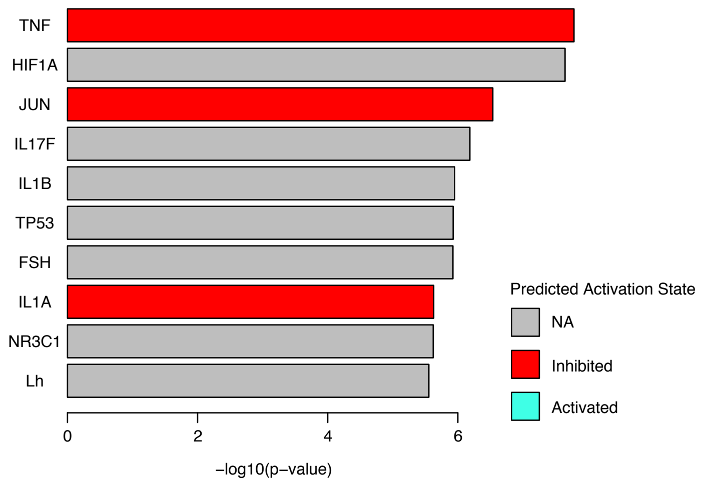

Supplementary Figure S3. Barplot of the top upstream regulators of the genes found to be differentially expressed between vehicle and R1881 exposed PCDFs.

Figure S4. CCL2 and CXCL8 effect on PCa cells proliferation

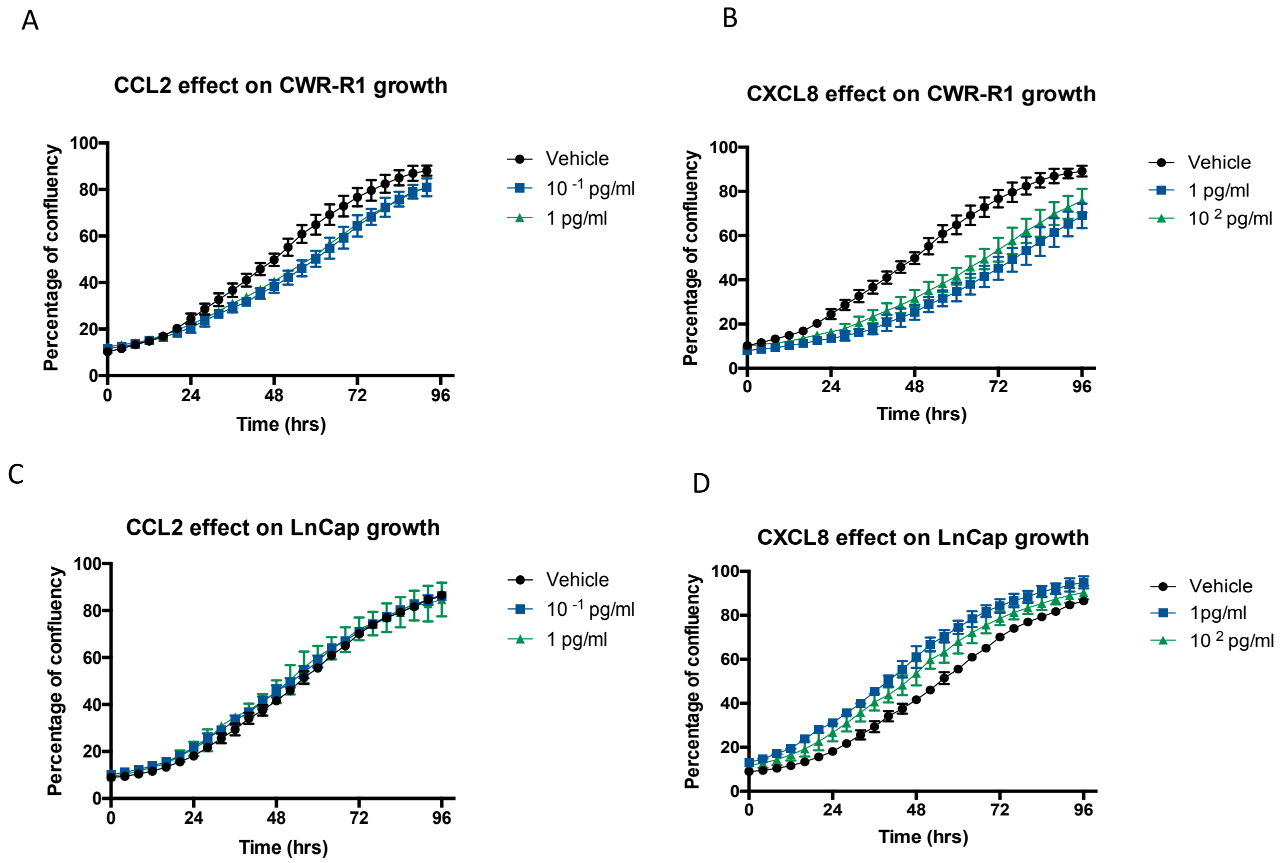

Supplementary Figure S4. Cell proliferation of the human prostate cancer cell lines CWR-R1 (A and B) and LNCaP (C and D) exposed to CCL-2 (MCP-1; 10-1 pg/ml and 1 –g/ml) and CXCL8 (IL-8; 1 pg/ml and 10-2 pg/ml). Average of 3 experiments, error bars indicate standard deviation

Figure S5. Migration and invasion assay

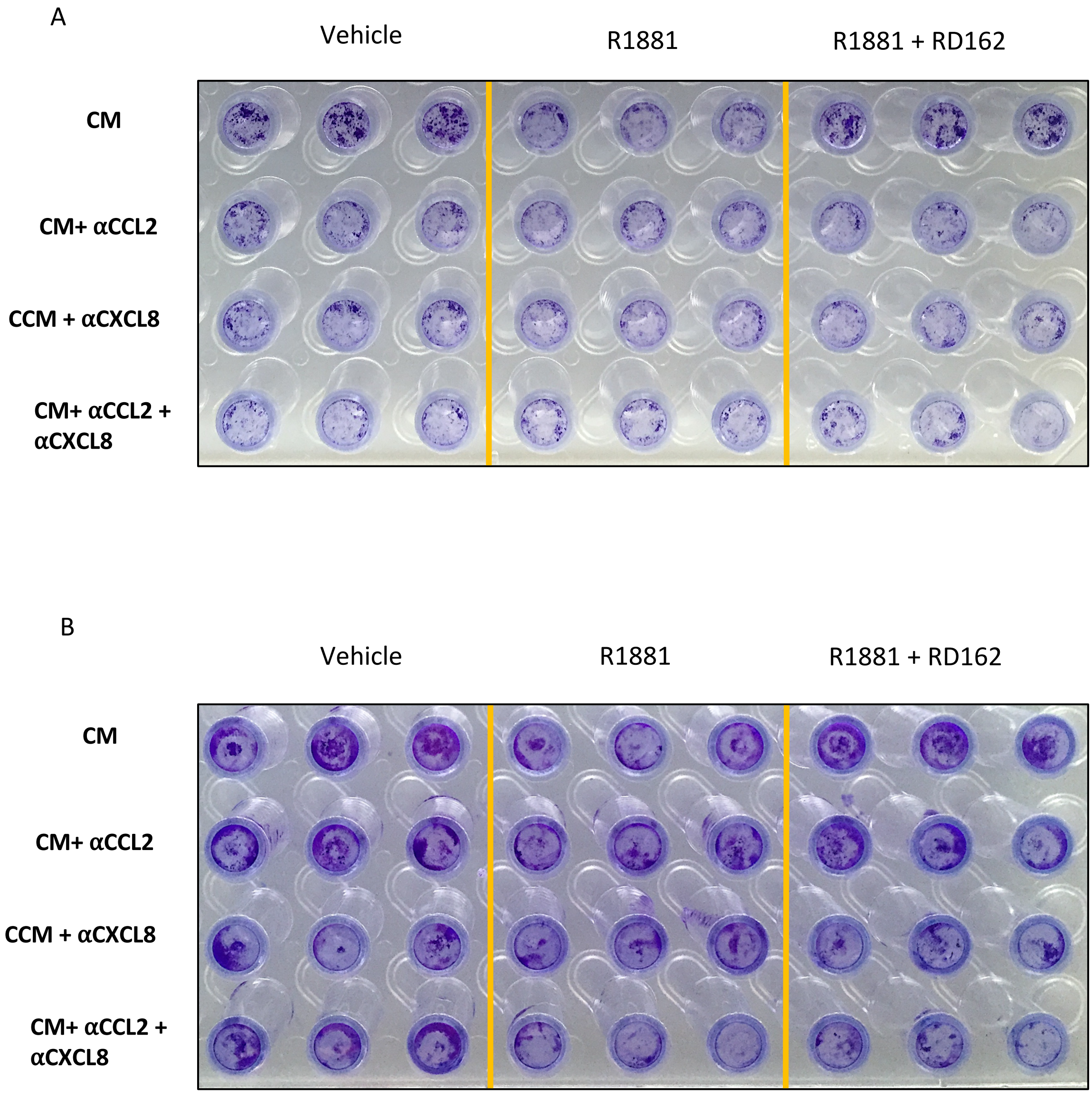

Supplementary Figure S5. Transwell assay was used to assess migration (A) and invasion (B) ability of CWR-R1 in fibroblast-conditioned medium (CM) with or without anti-CCL2 and/or anti-CXCL8 neutralizing antibody. Representative of two independent experiments.

Table S1. Antibodies list

Antibodies	Clone	Method
AMACR (Dako, Ely, UK)	13H4	IHC
AR (Cell Marque, Ventana Medical Systems, Tucson, AZ)	SP107	IHC
AR N20 (Santa Cruz Biotechnology)	SC-816	WB/ChIP
β-actin (Sigma)	AC-74	WB
H3K27Ac (Active Motif)	MABI 0309	ChIP
Pan-Cytokeratin (Thermo Fisher)	AE1AE3	IHC
PDGFRβ (AbCam, Cambridge, MA, USA)	Y92	WB
PSA (DAKO)	M0750	IHC
PSA (Cell Signaling Technology, Leiden, the Netherlands)	D11E1	WB
SMA-α (Thermo Fisher, Waltham, MA, USA)	1A4	WB
Vimentin (Chemicon)	ECB2	WB

Supplementary Table S1. List of antibodies used in the various experiments

Table S2. Culture media conditions

Supplements	Medium 1	Medium 2	Medium 3
DMEM (Dulbecco Modified eagle Medium)	✓		
RPMI1640 (Roswell Park Memorial Institute 1640)		✓	
DMEM/F-12			✓
4,5 g/l L D-Glucose and L-Glutamide	✓	✓	
10% FCS or 5% dextran- coated charcoal (DCC)	✓	✓	
100IU/ml penicillin	✓	✓	
100 µg/ml streptomycin	✓	✓	
1% bovine serum albumine (BSA)			✓
10 ng/ml Epidermal growth factor (EGF)			✓
1% Insulin / Transferin / Selenium (ITS)			✓
hydrocortisone 0,5 µg/ml			✓
1nM Triiodothyronine (T3)			✓
50ng/ml Cholera toxin			✓
20 µg/ml Fetuin			✓

Supplementary Table S2. Culture media used in our experiments

Table S3. R1881-specific upregulated genes found with BETA analysis

Chroms	txStart	txEnd	refseqID	rank product	Strands	GeneSymbol	FoldChange	p value
chr6	3,6E+07	35696360	NM_001145775	5,82E-06	-	FKBP5	1,51E-01	2,91E-02
chr11	8,5E+07	85469112	NM_001162951	6,68E-06	-	SYTL2	2,83E-01	1,20E-02
chr15	4,1E+07	41248717	NM_001142776	9,97E-06	+	CHAC1	1,44E-01	1,76E-02
chrX	1,1E+08	106960291	NM_004089	3,35E-05	-	TSC22D3	2,63E-01	6,10E-04
chr5	1,7E+08	172198203	NM_004417	3,95E-05	-	DUSP1	2,71E-01	3,82E-03
chr12	6603297	6641132	NM_014865	4,63E-05	+	NCAPD2	5,04E-01	1,04E-02
chr5	1,5E+07	14616287	NM_019018	5,16E-05	+	FAM105A	6,05E-01	2,34E-04
chr8	1,4E+08	145025044	NM_201380	8,15E-05	-	PLEC	1,75E-01	1,79E-02
chr16	6,7E+07	67323403	NM_015432	1,50E-04	+	PLEKHG4	3,54E-01	4,97E-04
chr12	6571403	6580065	NM_016830	1,54E-04	-	VAMP1	3,11E-01	3,40E-03
chr16	5,7E+07	56643409	NM_005953	1,77E-04	+	MT2A	1,58E-01	2,24E-02
chr11	6,6E+07	66104000	NM_004292	1,92E-04	-	RIN1	2,23E-01	2,86E-05
chr13	1,1E+08	114545814	NR_044995	2,20E-04	+	GAS6-AS1	1,25E-01	1,55E-02
chr10	1,3E+08	129924468	NM_002417	2,21E-04	-	MKI67	2,27E-01	1,82E-03
chr8	1,4E+08	142205900	NM_014957	2,23E-04	+	DENND3	5,75E-01	1,18E-04
chr16	2,2E+07	22300066	NM_013302	2,43E-04	+	EEF2K	1,84E-01	4,44E-04
chr19	4,2E+07	41767672	NM_001278599	2,71E-04	+	AXL	3,73E-01	5,35E-04
chr12	1,2E+08	123595036	NM_001300801	3,09E-04	-	PITPNM2	4,79E-01	1,62E-05
chr17	3,9E+07	39183454	NM_031957	3,55E-04	-	KRTAP1-5	2,12E-01	2,59E-03
chr5	1,8E+08	176981548	NM_001190946	4,06E-04	-	FAM193B	1,76E-01	2,28E-02
chr12	4,8E+07	48213763	NM_015401	4,06E-04	-	HDAC7	4,42E-01	1,00E-03
chr7	8,7E+07	87029112	NM_021151	4,64E-04	+	CROT	5,75E-01	2,47E-04
chr1	4,1E+07	40888998	NM_001198978	5,69E-04	+	SMAP2	5,96E-01	1,53E-03
chr12	7260903	7274447	NR_026947	5,75E-04	+	C1RL-AS1	2,44E-01	3,41E-04
chr12	5,6E+07	56230030	NR_036479	6,36E-04	+	TMEM198B	1,89E-01	6,04E-03
chr9	1,4E+08	136006544	NM_001271774	6,59E-04	-	RALGDS	4,77E-01	1,03E-02
chr8	3,9E+07	38831430	NM_021623	7,04E-04	+	PLEKHA2	2,19E-01	1,76E-03
chr19	1,6E+07	16346156	NM_001130524	7,08E-04	+	AP1M1	4,52E-01	4,23E-03
chr6	1,7E+08	170102159	NM_001202550	7,38E-04	-	WDR27	2,10E-01	1,33E-02
chr8	1,3E+08	126450647	NM_001282985	8,16E-04	+	TRIB1	4,74E-01	1,86E-04
chr15	6,5E+07	64673709	NM_001029989	8,61E-04	-	KIAA0101	2,42E-01	2,58E-02
chr1	1,6E+08	156107657	NM_001282624	8,89E-04	+	LMNA	6,89E-01	5,79E-04
chr12	4,9E+07	49222726	NM_001206917	9,47E-04	+	CACNB3	1,76E-01	2,51E-02
chr3	1E+07	10141344	NM_033084	9,73E-04	+	FANCD2	2,62E-01	3,78E-04
chr8	2,7E+07	27534286	NM_182826	9,82E-04	+	SCARA3	5,16E-01	5,56E-03
chr2	2,1E+07	20649204	NM_004040	1,02E-03	+	RHOB	9,63E-01	7,54E-11
chr19	1,9E+07	19223841	NM_178526	1,05E-03	+	SLC25A42	1,94E-01	1,63E-03
chr10	6,4E+07	63856707	NM_001244638	1,11E-03	+	ARID5B	1,39E-01	3,56E-02
chr1	5,4E+07	54199877	NM_147193	1,16E-03	-	GLIS1	1,90E-01	1,34E-02
chr18	2,1E+07	21376117	NR_130106	1,21E-03	+	LAMA3	4,07E-01	2,57E-04
chr17	8043787	8055753	NM_002616	1,22E-03	-	PER1	3,44E-01	5,39E-04
chr9	1,1E+08	106903700	NM_001265602	1,28E-03	+	SMC2	1,99E-01	2,85E-02
chr11	1,1E+08	112089649	NM_001256398	1,36E-03	+	BCO2	5,43E-01	1,25E-07
chr9	1,3E+08	127905838	NM_173690	1,36E-03	-	SCAI	3,90E-01	2,03E-03
chr9	1,2E+08	123657174	NM_001286840	1,37E-03	-	PHF19	1,76E-01	1,82E-02
chr3	1,3E+08	126076236	NM_014079	1,40E-03	-	KLF15	4,48E-01	3,10E-04
chr2	4,2E+07	42285668	NM_138370	1,48E-03	+	PKDCC	2,48E-01	6,97E-03

Supplementary Table S3. BETA analysis led to the identification of 174 statistically significant upregulated genes in CAF-like cells upon R1881 stimulation.

Table S4. R1881-specific downregulated genes found with BETA analysis

Chroms	txStart	txEnd	refseqID	rank product	Strands	GeneSymbol	FoldChange	p value
chr4	74606222	74609433	NM_000584	9,4E-06	+	CXCL8	-1,84E+00	4,22E-09
chr17	32582295	32584220	NM_002982	2,9E-05	+	CCL2	-1,10E+00	2,43E-10
chr11	102188180	102210135	NM_001165	3,8E-05	+	BIRC3	-1,32E+00	9,54E-11
chr4	177134825	177190373	NM_080874	5,0E-05	-	ASB5	-6,95E-01	8,17E-09
chr2	163027193	163100045	NM_004460	5,9E-05	-	FAP	-4,26E-01	2,34E-08
chr11	114166534	114183238	NM_006169	7,4E-05	+	NNMT	-3,56E-01	2,61E-07
chr11	44587140	44641315	NM_002231	8,2E-05	+	CD82	-2,88E-01	2,45E-04
chr17	74675650	74705679	NR_130926	8,2E-05	-	MXRA7	-2,66E-01	4,02E-06
chr4	86396283	86923823	NM_001025616	9,6E-05	+	ARHGAP24	-4,44E-01	1,07E-05
chr6	160102754	160114353	NM_000636	1,1E-04	-	SOD2	-1,02E+00	2,39E-09
chr12	7187514	7245043	NM_001733	1,1E-04	-	C1R	-6,56E-01	5,67E-10
chr12	16500711	16517344	NR_048547	1,4E-04	+	MGST1	-4,04E-01	2,16E-07
chr8	27454433	27472328	NM_001831	1,4E-04	-	CLU	-2,73E-01	3,46E-06
chr11	12183625	12285337	NM_001282666	1,5E-04	+	MICAL2	-1,13E-01	1,83E-02
chr11	9779839	9832866	NR_036485	1,7E-04	+	SBF2-AS1	-7,69E-01	4,13E-07
chr7	30791750	30797218	NM_001199219	1,7E-04	+	INMT	-6,05E-01	1,95E-06
chr12	51379774	51403053	NM_001174130	2,0E-04	-	SLC11A2	-2,16E-01	5,78E-04
chr14	35870715	35873960	NM_020529	2,0E-04	-	NFKBIA	-4,76E-01	6,25E-06
chr18	46446222	46469177	NM_001190823	2,1E-04	-	SMAD7	-2,00E-01	2,70E-03
chr10	124221040	124274424	NM_002775	2,2E-04	+	HTRA1	-3,99E-01	1,67E-08
chr15	40091222	40121477	NM_001271855	2,5E-04	-	GPR176	-2,28E-01	4,36E-05
chr18	47088426	47119278	NM_006033	2,5E-04	+	LIPG	-4,95E-01	9,73E-09
chr9	103340360	103350669	NM_001018116	2,5E-04	+	MURC	-5,84E-01	3,56E-03
chr6	123110193	123130864	NM_006714	2,7E-04	+	SMPDL3A	-5,90E-01	3,26E-08
chr2	46524540	46613842	NM_001430	2,7E-04	+	EPAS1	-5,31E-01	8,81E-09
chr21	30517879	30548210	NM_001286624	2,8E-04	+	MAP3K7CL	-3,51E-01	3,27E-04
chr8	11700033	11725646	NM_147782	3,0E-04	-	CTSB	-2,64E-01	6,85E-07
chr6	132617193	132722673	NM_015529	3,1E-04	-	MOXD1	-6,50E-01	3,47E-08
chr12	7167979	7178335	NM_001734	3,2E-04	+	C1S	-6,25E-01	1,29E-08
chr11	35453375	35551848	NM_001282675	3,3E-04	-	PAMR1	-5,42E-01	4,09E-08
chr7	129932973	129964020	NM_001163446	3,3E-04	+	CPA4	-2,78E-01	4,02E-03
chr2	162848754	162931052	NM_001935	3,7E-04	-	DPP4	-4,16E-01	2,09E-06
chr10	73576054	73611082	NM_002778	3,9E-04	-	PSAP	-4,08E-01	9,60E-08
chr3	112323232	112359990	NM_199512	4,1E-04	-	CCDC80	-3,44E-01	2,06E-07
chr1	153963238	153964631	NM_001030	4,2E-04	+	RPS27	-3,63E-01	4,23E-08
chr9	124101265	124132582	NM_004099	4,4E-04	-	STOM	-3,38E-01	2,11E-06
chr10	90973325	91011796	NM_000235	4,4E-04	-	LIPA	-4,47E-01	8,11E-08
chr9	103191795	103214016	NM_001198806	4,6E-04	+	MSANTD3	-1,41E-01	2,79E-02
chr5	80256507	80525981	NM_006909	4,6E-04	+	RASGRF2	-4,53E-01	2,48E-07
chr4	177604684	177713899	NM_005429	5,1E-04	-	VEGFC	-2,88E-01	1,39E-05
chr9	101705994	101833074	NM_001855	5,6E-04	+	COL15A1	-5,01E-01	6,73E-08
chr15	89441913	89456663	NM_005928	5,6E-04	-	MFGE8	-2,66E-01	4,18E-06
chr13	36875774	36920934	NM_001142295	6,1E-04	-	SPG20	-4,34E-01	2,02E-07
chr12	10851675	10875953	NM_001145426	6,3E-04	-	YBX3	-2,74E-01	1,41E-05
chr11	93862093	93915137	NM_015368	6,4E-04	+	PANX1	-3,82E-01	6,04E-07
chr20	48120410	48184707	NM_000961	7,1E-04	-	PTGIS	-7,60E-01	1,89E-07
chr1	110546569	110566364	NM_001242676	7,5E-04	+	AHCYL1	-1,00E-01	1,64E-02

Supplementary Table S4. BETA analysis led to the identification of 234 statistically significant downregulated genes in CAF-like cells upon R1881 stimulation.
